# Is the New Interferon-Gamma Releasing Assay Beneficial for the Diagnosis of Latent and Active *Mycobacterium tuberculosis* Infections in Tertiary Care Setting?

**DOI:** 10.3390/jcm10071376

**Published:** 2021-03-29

**Authors:** Jaewan Jung, Byung Woo Jhun, Mijeong Jeong, Sun Joo Yoon, Hee Jae Huh, Chul Won Jung, Kihyun Kim, Jae Berm Park, Dae Joong Kim, Wooseong Huh, Hye Ryoun Jang, Young-Ho Kim, Sung Noh Hong, Doo Ryeon Chung, Eun-Suk Kang

**Affiliations:** 1 Department of Laboratory Medicine and Genetics, Samsung Medical Center, Sungkyunkwan University School of Medicine, Seoul 06351, Korea; halfleaf@naver.com (J.J.); yoonsunjoo@gmail.com (S.J.Y.); heejae.huh@samsung.com (H.J.H.); 2 Division of Pulmonary and Critical Care Medicine, Samsung Medical Center, Sungkyunkwan University School of Medicine, Seoul 06351, Korea; byungwoo.jhun@samsung.com; 3 Research Institute for Future Medicine, Samsung Medical Center, Sungkyunkwan University School of Medicine, Seoul 06351, Korea; mj81.jeong@sbri.co.kr; 4 Division of Hematology-Oncology, Samsung Medical Center, Sunghyunkwan University School of Medicine, Seoul 06351, Korea; chulwon1.jung@samsung.com (C.W.J.); kihyunk.kim@samsung.com (K.K.); 5 Department of Transplantation Surgery, Samsung Medical Center, Sungkyunkwan University School of Medicine, Seoul 06351, Korea; jaeberm.park@samsung.com; 6 Division of Nephrology, Department of Medicine, Samsung Medical Center, Sungkyunkwan University School of Medicine, Seoul 06351, Korea; daejoongsmc.kim@samsung.com (D.J.K.); wooseong.huh@samsung.com (W.H.); hyeryoun.jang@samsung.com (H.R.J.); 7 Division of Gastroenterology, Department of Medicine, Samsung Medical Center, Sungkyunkwan University School of Medicine, Seoul 06351, Korea; yhgi.kim@samsung.com (Y.-H.K.); sungnoh.honb@samsung.com (S.N.H.); 8 Division of Infectious Diseases, Department of Medicine, Samsung Medical Center, Sungkyunkwan University School of Medicine, Seoul 06351, Korea; dr.chung@samsung.com; 9 Stem Cell & Regenerative Medicine Institute Research, Institute for Future Medicine, Samsung Medical Center, Sungkyunkwan University School of Medicine, Seoul 06351, Korea

**Keywords:** *Mycobacterium tuberculosis*, IGRA, interferon-gamma, active tuberculosis, LTBI, intracellular cytokine staining

## Abstract

Interferon-Gamma Release Assays (IGRAs) are widely used in the laboratory diagnosis of *Mycobacterium tuberculosis* (MTB) infections, particularly in the latent form. We compared the performance of a newly developed IGRA, the Standard E TB-Feron ELISA (TBF) with the currently used QuantiFERON-TB Gold Plus assay (QFT-Plus) for the detection of latent tuberculosis infections (LTBIs) in tertiary care settings. We also investigated interferon-gamma (IFN-γ) released by T cell subsets via intracellular cytokine staining (ICS) and flow cytometry. A total of 335 subjects including 40 patients with active tuberculosis (ATB), 75 immunocompromised patients with LTBIs (P-LTBI), 70 health care workers with LTBIs (H-LTBI), and 150 healthy controls (HC) were studied. Overall, 168 subjects (50.1%) and 178 subjects (53.1%) displayed IGRA-positive results in the QFT-Plus and TBF, respectively. The overall concordance rate was 94.0%. The sensitivity and specificity of TBF were 88% and 95%, respectively, while the sensitivity and specificity of QFT-Plus were 90% and 100%, respectively. Twenty discordant results (6.0%) were observed in simultaneously performed QFT-Plus and TBF. Particularly, 13 LTBI subjects previously positive QFT-Plus showed negative results in QFT-Plus performed after enrollment. In TBF, six subjects showed positive results while five were negatively concordant with QFT-plus and two were indeterminate. The overall proportion of IFN-γ releasing CD8+ T lymphocytes was significantly higher in TBF compared to those of QFT-Plus TB1 and TB2 (0.21% vs. 0.01% and 0.02%; *p*-value < 0.05). The recombinant protein antigens in the TBF stimulated TB-specific CD8+ T cells more efficiently. Therefore, TBF would be a useful alternative to current IGRAs such as the QFT-Plus, particularly in tertiary care settings where the immunocompromised patients are subjected to IGRA tests to differentiate MTB infection. Further strategies to analyze the implications of the discrepancies, particularly near the cutoff values between different IGRAs, are needed.

## 1. Introduction

Tuberculosis (TB) remains one of the leading infectious diseases worldwide. The Republic of Korea carries an intermediate burden of *Mycobacterium tuberculosis* (MTB), with 23,821 new cases and an incidence of 46.4 per 100,000 people detected in 2019. Although the number of new TB cases decreased compared to the rate of 51.5 per 100,000 people reported in 2018, the proportion of patients aged 65 years or older increased from 45.5% in 2018 to 47.1% in 2019 [1,2]. Thus, a substantial national effort is needed to manage TB infections. However, the diagnosis of TB infections using existing tools is not satisfactory due to the low sensitivity of smear microscopy, the long duration of cultures, and inconclusive results of immunologic assays [3,4].

In the last two decades, Interferon-Gamma Release Assays (IGRAs) have been developed and used to diagnose latent TB infections (LTBIs), and as alternatives to tuberculin skin test (TST) with the advantages of minimally invasive ex vivo blood tests and no influence by Bacillus Calmette–Guérin (BCG) strains such as *M. bovis* and most atypical mycobacterial infections [5,6,7]. IGRAs measured T cell response after stimulation with antigens specific for the MTB complex such as early secreted antigenic target 6 (ESAT-6), culture filtrate protein 10 (CFP-10), and TB7.7 [5,6]. LTBI is not transmitted and has no evidence of clinical symptoms or signs of active TB (ATB). However, nearly 5–15% of individuals with LBTI eventually develop active tuberculosis [8,9]. Further, immunocompromised patients and those treated with immunosuppressive drugs for various diseases are at an increased risk of opportunistic infections such as MTB [10]. Because the risk of progression to ATB can be significantly reduced by preventive TB medication [11], the identification of individuals with LTBIs is an important component of TB treatment and elimination from the perspective of public preventive medicine and patient care.

Considering no gold standard test for the diagnosis of LTBIs, both IGRAs and TSTs only provide indirect information related to LTBIs [12]. Further, the sensitivity of IGRA is lower in immunocompromised patients and children than in healthy adult individuals [13,14,15] and is poorly correlated with active disease [16]. 

The QuantiFERON-TB Gold In-Tube assay (QFT-GIT; Qiagen, Germantown, MD), an enzyme-linked immunosorbent assay (ELISA), is the most commonly used IGRA to identify LTBIs. The QFT-GIT is designed to stimulate CD4+ T lymphocytes to release interferon-gamma (IFN-γ) in a single TB antigen tube containing long peptides from three TB antigens (ESAT-6, CFP-10, and TB7.7). Within the past few years, the QuantiFERON-TB Gold Plus assay (QFT-Plus; Qiagen, Germantown, MD), which is a new-generation QFT-GIT, has been used as a substitute for the QFT-GIT. Several studies comparing the QFT-Plus and QFT-GIT showed equivalent sensitivity and high overall agreement in subjects with recent contact with ATB patients, in healthcare workers under low TB incidence settings, and immunocompromised patient populations [17,18,19].

In 2019, the Standard E TB-Feron ELISA (TBF; SD Biosensor, Gyeonggi-do, Korea) was newly introduced and approved by the Ministry of Food and Drug Safety of the Republic of Korea. The TBF is also available in a single TB antigen tube format similar to the QFT-GIT, but the TBF and QFT differ predominantly in the TB-specific antigens. In the TBF, the antigens are recombinant whole proteins of ESAT-6, CFP-10, and TB7.7 in contrast to recombinant peptide mixtures in the QFT-GIT and QFT-Plus [20,21]. A recent study showed comparable results TBF and QFT-GIT and acceptable clinical performance for detecting LTBIs in healthcare workers [7]. However, no study has compared the TBF and QFT-Plus results in patient populations until now. 

The purpose of this study was to compare the diagnostic performance of TBF, the new IGRA test, to that of QFT-Plus and investigate whether the new IGRA test had the benefit of detecting LTBIs in tertiary care settings where the majority of IGRA tests were being performed in immunocompromised patients. 

## 2. Methods

### 2.1. Patients and Controls

The study was performed from November 2018 to January 2020 at the Samsung Medical Center (Seoul, Korea), a tertiary care referral hospital. All participants were adults aged above 18 years and were divided into four groups: ATB, P-LTBI (patients with immunocompromised status and LTBIs), H-LTBI (health care workers (HCWs) with LTBIs), and HC (HCWs confirmed with previous negative IGRA results and low risk). The inclusion criteria for each group were as follows: the ATB group had 1) a positive MTB isolation culture involving solid or liquid media and/or 2) MTB-polymerase chain reaction (PCR), and 3) were newly diagnosed TB patients with none or less than four weeks of anti-TB therapy. The ATB patients in the early treatment phase were included in this study based on previous reports that IFN-γ levels of QFT-Gold started to decrease from 4 weeks and functional T cells were significantly affected at 8 weeks after the initiation of treatment in pulmonary TB patients [22,23]. The P-LTBI group included patients who tested positive in a previous IGRA and carried a high risk of LTBI reactivation based on scheduled interventions (such as kidney and liver transplantation due to end-stage renal disease and hepatocellular carcinoma, respectively, or hematopoietic stem cell transplantation due to underlying malignancy and chemotherapy for the management of leukemia, lymphoma, and plasma cell myeloma, and scheduled to receive immunosuppressive therapy for systemic autoimmune diseases, such as Behcet’s disease, Crohn’s disease, and ulcerative colitis). HCWs in the H-LTBI group had known positive IGRA results without active TB symptoms and had no other previous medical history including anti-TB medication except LTBI. The HC group consisted of healthy volunteers among HCWs without the risk of TB exposure. The risk of TB exposure in the HC group was investigated via a questionnaire survey of previous TB history, family history of TB, personal contact with TB patients, and working in hospital departments with a high risk of TB exposure. Written informed consent was obtained from all participants. The study protocol was approved by the Samsung Medical Center Ethics Review Committee (Institutional Review Board no. 2018-08-128).

### 2.2. QFT-Plus and TBF Assays

The QFT-Plus assays were performed according to the manufacturer’s instructions. Whole blood collected in lithium-heparin tubes was distributed (with 1 mL of whole blood in each tube) into TB antigen tubes 1 and 2 (TB1 and TB2), mitogen tube, and nil tube for both assays. After 20 h of incubation at 37 °C, the tubes were centrifuged for 15 min at 2000× g and the plasma was separated. An ELISA kit was used to quantify the IFN-γ in the plasma samples. The results were interpreted as positive when the IFN-γ value for any of TB antigen tube (TB antigen value) minus that of the nil tube (nil value), referred to as the TB antigen-minus-nil value, was 0.35 IU/mL and increased by at least 25% of the nil value. If the nil value was 8.0 IU/mL or the value of the mitogen tube (mitogen value) minus the nil tube value was <0.50 IU/mL, the results were considered indeterminate. Any other results were deemed negative.

The TB-Feron assays were performed according to the manufacturer’s instructions. Whole blood collected in lithium-heparin tubes was added (with 1 mL of whole blood in each tube) to the three tubes: the TB antigen, nil, and mitogen tubes. After 20 h of incubation at 37 °C, the tubes were centrifuged for 15 min at 2000× g and the plasma was separated. The ELISA kit was used to quantify the IFN-γ in the plasma samples. The results were interpreted as positive when the IFN-γ value of the TB antigen tube (TB antigen value) minus that of the nil tube (nil value), referred to as the TB antigen-minus-nil value was 0.35 IU/mL and at least 25% of the nil value. The results were considered indeterminate if the nil value was 8.0 IU/mL or the value of the mitogen tube (mitogen value) minus the nil value was <0.50 IU/mL. Any other results were deemed negative. The results from the patients (ATB group) and controls (HC group) were used to calculate the sensitivity and specificity for the diagnosis of active TB. 

In case of discordant results, the previous IGRA results (QFT-GIT/QFT-Plus assays) were analyzed, and if the subjects agreed and their condition did not change significantly, QFT-Plus and TBF were repeated.

### 2.3. Intracellular IFN-γ Cytokine Analysis

Intracellular cytokine staining (ICS) was performed concomitantly with the QFT-Plus and TBF. Peripheral blood mononuclear cells (PBMCs) were isolated using Ficoll (GE Healthcare, Chicago, IL, USA) density gradient centrifugation and resuspended in complete RPMI-1640 medium (Welgene, Kyeongsan-kun, Korea) with 10% fetal bovine serum (ThermoFisher Scientific, Waltham, MA, USA) (R10 media).

To characterize the MTB-specific T cell response via flow cytometry, 1 × 10^6^ PBMCs resuspended in 1 mL of Gibco^®^ RPMI 1640 (ThermoFisherScientific, Waltham, MA, USA) containing 10% fetal bovine serum (ThermoFisherScientific, Waltham, MA, USA) (R10) were dispensed into each TB1, TB2, and mitogen tubes of the QFT-Plus kit, and the nil, TB, and mitogen tubes of the TBFeron kit. After thoroughly mixing and incubating at 37 °C for 5 min, the PBMCs were transferred to new sets of QFT-Plus and TBF tubes to allow sufficient contact between the PBMCs and the peptides. After incubation for an hour at 37 °C, the PBMCs were transferred to polystyrene round-bottom tubes and cultured with anti-CD28 (ThermoFisherScientific, Waltham, MA, USA) and anti-CD49d (R&D Systems, Minneapolis, MN, USA) monoclonal antibodies (mAb) at a concentration of 2 mg/mL each and 1 µg/mL of Golgi plug (BD Biosciences, San Jose, CA, USA) as described previously [2,20,21,22,23]. ICS was performed following incubation at 37 °C for 16 to 24 h. As previously described, the PBMCs were stained with anti-CD4 (clone: RPA-T4) Alexa Fluor^®^ 488 conjugate (Biolegend, San Diego, CA, USA), anti-CD8 (clone: SK1) APC-H7 conjugate, and anti-CD3 (clone: UCHT1) PerCp-Cy5.5 conjugate (BD Biosciences, San Jose, CA, USA). After fixation and permeabilization using IntraPrep (Beckman Coulter, Brea, CA, USA), the cells were stained with anti-IFN- (clone: 4S.B3) PE conjugate (ThermoFisherScientific, Waltham, MA, USA). The TB-specific T cell response was characterized by evaluating the frequency of IFN-γ secreting CD4+ and IFN-γ secreting CD8+ T lymphocytes. At least 100,000 lymphocytes were acquired with a FACS CANTO II (BD Biosciences, San Jose, CA, USA). The cytometry data were analyzed using BD FACSDiva software version 8.5 (BD Biosciences, San Jose, CA, USA). The background cytokine synthesis in the TBF nil tube was subtracted from each stimulated condition. The results were scored as absence of response if the background was higher than the antigen-specific response.

### 2.4. Statistical Analysis

The accordance between QFT-Plus and TBF, and their results, which were performed simultaneously, were assessed using Cohen’s kappa coefficients and interpreted according to the criteria proposed by Landis and Koch [24,25]: bad scores, 0.01 to 0.20; fair scores, 0.21 to 0.40; moderate scores, 0.41 to 0.60, strong scores, 0.61 to 0.80, and almost perfect scores, 0.81 to 1.00. Quantitative variables were expressed as the median, interquartile range (IQR), range, and 95% confidence interval (CI) and analyzed using the Wilcoxon signed-rank test. The data were analyzed using IBM SPSS statistics (version 22.0) software (IBM Corporation, Armonk, NY, USA). All reported *p*-values were two-tailed and calculated with statistical significance set at a *p*-value of less than 0.05.

## 3. Results

### 3.1. Subject Characteristics

**Figure 1:**
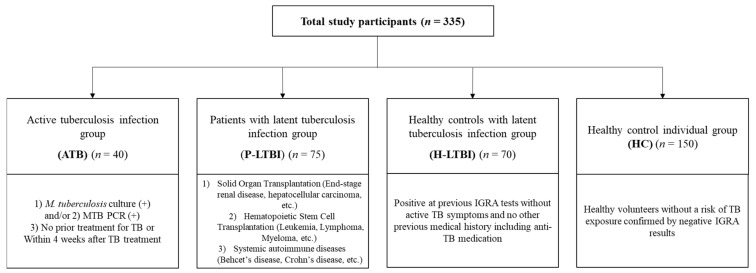
Study group and inclusion criteria. Four groups were included; ATB, patients diagnosed active tuberculosis; P-LTBI, patients with immunosuppressive status diagnosed latent tuberculosis infection; H-LTBI, Healthy controls with diagnosed latent tuberculosis infection; HC, healthy control individuals with minimal risk to tuberculosis (TB) exposure. Abbreviation: MTB-PCR, Mycobacterium tuberculosis polymerase chain reaction.

A total of 335 subjects were included in this study. The median age of the subjects was 39 years (interquartile range [IQR] 30–54), and males constituted 38.2% (128/335) of the subjects. Forty active TB cases including seven (17.5%) isolated extra-pulmonary TB patients were enrolled in the ATB group. We enrolled 75 LTBI patients with immunosuppressive conditions in the P-LTBI group and 70 HCWs with known IGRA-positive results without active TB symptoms and other medical history including anti-TB medication in the H-LTBI group. The HC group included 150 healthy HCWs or volunteers without risk of TB exposure. Characteristics of four groups and their inclusion criteria are summarized in Table 1 and Figure 1.

### 3.2. Agreement of QFT-Plus and TB-Feron Results

In total, 335 subjects were tested with the QFT-Plus and TBF assays. Overall, 168 subjects (50.1%) and 178 subjects (53.1%) displayed IGRA-positive results in the QFT-Plus and TBF, respectively. Table 2 summarizes the QFT-Plus and TBF results in each group. Among 168 subjects with positive results based on QFT-Plus, 161 (95.8%) tested positive in both TB1 and TB2, but two (1.2%) and five (3.0%) subjects were positive only in TB1 (TB1+TB2-) and TB2 (TB1-TB2+), respectively. The overall concordance between the QFT-Plus and TBF was 94.0% (κ value = 0.892) and the concordance rate within each ATB, P-LTBI, H-LTBI, and HC group was 92.5%, 90.7%, 95.7%, and 95.3%, respectively. The sensitivity of the TBF in the ATB group was 88% (35/40) and the specificity calculated in the HC group was 95% (143/150), while the sensitivity and specificity of the QFT-Plus were 90% (36/40) and 100% (150/150), respectively (Table 2).

A total of 25 subjects (7.5%) had inconsistent or discordant IGRA results with previously or simultaneously performed QFT-Plus and/or TBF (Table 3) except repeat IGRA during follow up. Twenty discordant results (6.0%) were observed in simultaneously performed QFT-Plus and TBF, which included three from ATB, seven from P-LTBI, three from H-LTBI and seven from HC.

**Figure 2:**
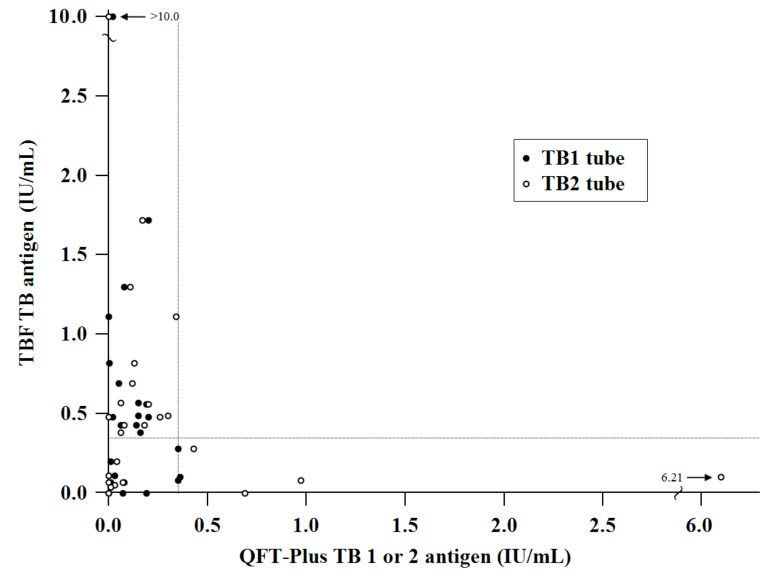
Scatter plot of IFN-γ concentrations in 25 cases with discrepant results between the TBF and QFT-Plus tests. Cut-off of both tests: IFN-γ 0.35 IU/mL (dotted line). Abbreviation: IFN-γ, interferon-gamma; TBF, Standard E TB-Feron ELISA; QFT-Plus, QuantiFERON-TB Gold Plus; TB, tuberculosis.

Particularly, among the LTBI group, 13 LTBI subjects who had previously positive QFT-GIT/QFT-Plus showed negative results in initial QFT-Plus performed after enrolment. In simultaneously performed TBF, six subjects (five P-LTBI and one H-LTBI) showed positive results while five subjects (P-LTBI) were negative as concordant with QFT-plus, and two subjects (P-LTBI) were indeterminate. In contrary, two H-LTBI had consistently positive results in QFT-Plus but were negative in TBF. In the HC group, seven subjects showed positive results in TBF. To further analyze the discordant cases, the QFT-Plus and TBF assays were repeated when possible, and seven cases were available. Among them, four cases had the same results with initial QFT-Plus and TBF, but three HC cases became negative from initially positive TBF. The IFN-γ values of 25 discordant results are plotted in Figure 2.

The overall IFN-γ values in the 25 discordant cases, including 20 cases between QFT-Plus and TBF and 5 cases between QFT-GIT/QFT-Plus and QFT-Plus, had median values of 0.08 IU/mL (range, <0 to 0.36 IU/mL, IQR 0.02–0.19 IU/mL), 0.18 IU/mL (range, <0 to 6.21 IU/mL, IQR 0.03–0.27 IU/mL) and 0.38 IU/mL (range, <0 to >10 IU/mL, IQR 0.08–0.60 IU/mL) in the QFT-Plus TB1, TB2 and TBF, respectively. Among 79 IFN-γ values of 25 discordant cases between simultaneously performed QFT-Plus and TBF, or between previous QFT-GIT/QFT-Plus and initial QFT-Plus listed in Table 2, 38.0% occurred between 0.31 and 1.00 IU/mL with median 0.33 IU/mL and IQR 0.11 to 0.55 IU/mL.

### 3.3. Intracellular Cytokine Analysis of IFN-γ-Secreting T Lymphocytes

**Figure 3:**
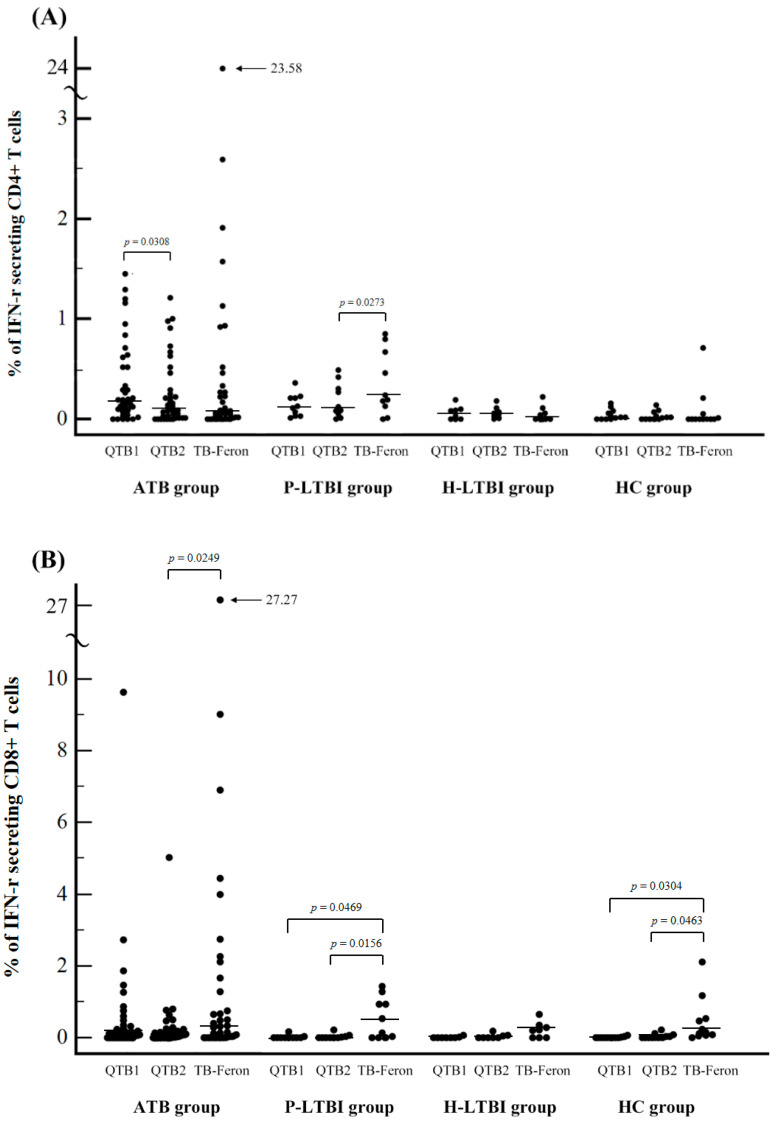
Distribution of TB antigen-specific IFN-γ secreting T cells in response to TB antigens in the QFT-Plus and TB-Feron assays measured using flow cytometry intracellular cytokine analysis. (**A**) CD4+ T cells (**B**) CD8+ T cells; Abbreviation: TB, tuberculosis; IFN-γ, interferon-gamma; QTB1, QFT-Plus TB1 tube, QFT2, QFT-Plus TB2 tube.

We next performed ICS to evaluate the direct cytokine release from CD4+ and CD8+ T lymphocytes in a single cell level in order to investigate whether the differences in antigenic stimulants of QFT-Plus and TBF could explain some of the different results. A total of 56 samples obtained from 38 participants in the ATB group, 10 in the P-LTBI, eight in the H-LTBI, and 12 in the HC group were included. The frequency of IFN-γ secreting CD4+ and CD8+ T cells per total lymphocytes is plotted in Figure 3A,B, respectively.

Overall, the median proportions of IFN-γ-releasing CD4+ T cells were 0.10% (95% CI: 0.07 to 0.16%), 0.05% (95% CI: 0.02 to 0.09%), and 0.06% (95% CI: 0.01 to 0.11%) for QFT-Plus TB1, TB2, and TBF, respectively. The median proportions of IFN-γ-releasing CD8+ T cells were 0.01% (95% CI: 0.00 to 0.07%), 0.02% (95% CI: 0.00 to 0.05%), and 0.21% (95% CI: 0.08 to 0.39%) for QFT-Plus TB1, TB2 and TBF, respectively. The proportions of IFN-γ-releasing CD8+ T cells of TBF were significantly increased versus those of QFT-Plus in all ATB, P-LTBI and HC groups. In P-LTBI group, the proportions of IFN-γ-releasing CD4+ T cells of TBF were also significantly increased versus those of QFT-Plus. Between TB1 and TB2 of QFT-Plus, there was no significant difference except IFN-γ-releasing CD4+ T cells in ATB group (Figure 3 and Table 4).

## 4. Discussion

In this study, we evaluated the performance of a newly developed IGRA, Standard E TB-Feron ELISA (TBF; SD Biosensor, Gyeonggi-do, Korea) for the diagnosis of MTB in the Republic of Korea, a country with an intermediate TB burden. TBF showed excellent agreement with QFT-Plus in different populations including immunocompromised patients while the variation was still notable near the IFN-γ cutoff values.

The diagnostic performance of the new IGRA kits was validated and compared with previous IGRA or TST results and other clinical evidence. The recently introduced QFT-Plus assay, based on a two-tube format with an additional target region, showed comparable performance with the QFT-GIT in diagnosing LTBIs. Moon et al. [19] reported a 95.6% overall agreement between the QFT-GIT and the QFT-Plus in 989 low-risk healthcare workers. Ryu et al. [24] compared the QFT-Plus and QFT-GIT for the diagnosis of LTBIs among 317 immunocompromised patients, and the rate of concordance between the assays was 93.7% (κ value, 0.860). To date, only one study has evaluated the TBF assay in the Republic of Korea by Kweon et al. [7], who compared the QFT-Plus with the newly introduced TBF kit for the diagnosis of LTBIs among 425 HCWs. The TBF demonstrated 81.6% positive and 97.4% negative concordance with the QFT-GIT (κ value, 0.780) in HCWs. In this study which included different conditions of MTB infection and various populations including immunocompromised patients, we found that the overall concordance rate between the QFT-Plus and TBF was 94.0% (κ value = 0.894) and the κ value between the two IGRAs represented a correlation similar to that of other related studies.

Although IGRA is known to be the standard practice in LTBI diagnosis but not in ATB diagnosis, the sensitivity of IGRAs has been estimated in laboratory-confirmed ATB due to a lack of confirmatory tests in LTBI. From that perspective, in our study, the sensitivity and specificity of the tests in detecting active TB infection were evaluated in the ATB group and HC group, respectively. Bae et al. [25] reported an overall sensitivity of 80.2% in the QFT-GIT for active TB infection diagnosis in the Korean population. Takasaki et al. [17] reported sensitivity and specificity for active TB of QFT-Plus, QFT-GIT and T-SPOT TB within the range of 96.9% to 99.1% in laboratory-confirmed active TB patients and healthy volunteers in Japan. Petruccioli et al. [20] reported sensitivities of QFT-Plus and QFT-GIT of 90% and 88%, respectively, for ATB cases and a specificity of 100% involving both QFT-Plus and QFT-GIT in low TB risk populations. Horne et al. [26] reported sensitivities of 94% and 93% with QFT-Plus and QFT-GIT, respectively, for ATB detection in 164 ATB patients. In our study, the TBF showed a slightly lower sensitivity than the QFT-Plus (88% vs. 90%) and the estimated specificity based on the HC group was lower than that of the QFT-Plus (95% vs. 100%), which were comparable with previous reports.

For further examination of the diagnostic ability, we analyzed 25 discrepant cases between the QFT-Plus and TBF. In previous studies investigating the variability and reproducibility of the QFT-GIT or QFT-Plus, the discordant results were mostly within the gray zone (TB antigen-minus-nil value, 0.2 to 0.7 IU/mL) [24,27,28,29]. Overall, 38.0% of discordant results between simultaneously performed QFT-Plus and TBF or between previous QFT-GIT/QFT-Plus and initial QFT-Plus occurred between 0.31 and 1.00 IU/mL in this study. These results verified a relatively poor concordance and reproducibility of IFN-γ values near the gray zone between different IGRA kits and different measuring time points in the same individuals, respectively. The agreement of QFT-Plus and TBF was the lowest in the LTBI group, particularly in the P-LTBI group versus others. Among eight subjects who had discrepant results between simultaneously performed QFT-Plus and TBF, five P-LTBI and one H-LTBI showed negative QFT-GIT/QFT-Plus which was inconsistent with previously positive QFT-GIT/QFT-Plus results. Although the repeat IGRA could have been done in three LTBI subjects, the results did not change. This might indicate that the immunocompromised status of the P-LTBI group affected the negative conversion of initial QFT-Plus as time goes on. It needs to be further elucidated whether using TBF is beneficial to overcome the effect of immune variation particularly in the P-LTBI group with serial tests including a large number of patients, although our result provided some evidence. There were seven HC who showed positive TBF and among four of them who subjected to repeat IGRA, three HC became negative TBF while all were persistently negative in QFT-Plus. Nevertheless, the potential false positivity of TBF in seven HC subjects affected the reduced specificity of TBF.

Since CD8+ T lymphocytes are known to play an important role in host immunity to TB and secrete IFN-γ via activation of macrophages using Fas receptors [30,31], the detection of IFN-γ released from CD8+ T lymphocytes may have contributed to the increased sensitivity of TB detection in the early phase. The QFT-Plus involved additional stimulants in the TB2 tube, while the TB1 tube was identical to the QFT-GIT except for the absence of the TB7.7 antigen. The TB2 tube contained a cocktail of six short peptides, in addition to the same components in the TB1 tube eliciting IFN-γ release from both CD4+ and CD8+ T lymphocytes. Nevertheless, no direct evidence was available to show that the recombinant TBF protein structure stimulated either CD4+ or CD8+ T lymphocytes effectively.

Therefore, in addition to indirectly measuring IFN-γ using the IGRA, we evaluated the direct cytokine release from CD4+ and CD8+ T lymphocytes in a single cell level via intracellular cytokine analysis using flow cytometry in order to investigate whether the differences in antigenic stimulants of QFT-Plus and TBF could explain some of the different results in different groups. In our study, the levels of IFN-γ-secreting T lymphocytes were significantly higher in the TBF compared to the QFT-Plus, especially from CD8+ T cells in all groups. Petruccioli et al. [32] reported higher CD8+ T cell responses in the TB2 tube of the QFT-Plus in patients with severe TB compared to patients with less severe TB, which suggested that CD8+ T cells might actively contribute to anti-TB immune responses in vivo. The TB antigens in TBF appeared to stimulate T cells, particularly CD8+ T cells more strongly compared to those of the QFT-Plus. This phenomenon might be an advantage in the P-LTBI group of patients who were immunocompromised although strong stimulation of T cells may increase the number of false-positive results in the low-risk healthy control group, which might explain the potential false positive results of TBF in HC group in this study. Nevertheless, in a tertiary care hospital setting where the IGRA is used in LTBI screening of many immunocompromised patients, TBF might show a higher sensitivity in capturing LTBI patients who were missed by other IGRAs. 

This study had a limitation to evaluate the performance of different IGRA assays in the LTBI group because it can be evaluated by long-term follow-up data in the absence of standardized tests for the diagnosis of LTBIs. The number of cases subjected to repeated IGRAs was not enough to estimate the variability affecting discordant results between QFT-Plus and TBF. 

In conclusion, the TBF showed diagnostic ability comparable to that of QFT-Plus in different groups including ATB, LTBI and HC subjects. Therefore, TBF would be a useful alternative to current IGRAs such as the QFT-Plus, particularly in tertiary care settings where the immunocompromised patients are subjected to IGRA tests to differentiate MTB infection. Because discordant results between different IGRAs persist, further strategies to analyze the implications of the discrepancies, particularly near the cutoff values, are needed.

## Figures and Tables

**Table 1:** Characteristics of study groups.

	Study Groups (*n* = 335)			
Characteristic	ATB (*n* = 40)	P-LTBI (*n* = 75)	H-LTBI (*n* = 70)	HC (*n* = 150)
Median (IQR) age (yr)	53 (41–63)	57 (51–63)	43 (36–52)	30 (26–34)
Male subjects, No. (%)	23 (57.5)	47 (62.7)	25 (35.7)	31 (20.7)
*M. tuberculosis* culture and PCR result, No. (%)				
Culture positive/PCR positive	9 (22.5)	NA	NA	NA
Culture negative/PCR positive	8 (20.0)	NA	NA	NA
Culture positive/PCR negative	23 (57.5)	NA	NA	NA
Manifestation of ATB, No. (%)				
Active pulmonary disease with or without extra-pulmonary TB	33 (82.5)	NA	NA	NA
Solely extra-pulmonary TB (Lymph node, colon nasopharynx, soft tissue, bone)	7 (17.5)	NA	NA	NA
Previous anti-tuberculosis medication, No. (%)	27 (67.5)	0 (0.0)	0 (0.0)	0 (0.0)
Median duration (range) of TB treatment before blood sampling, days (range)	12 (2–30)	NA	NA	NA
Underlying conditions, No. (%)				
Solid organ failure (liver, kidney)	0 (0.0)	37 (49.3)	0 (0.0)	0 (0.0)
Hematologic malignancy	0 (0.0)	6 (8.0)	0 (0.0)	0 (0.0)
Systemic autoimmune disease	0 (0.0)	32 (42.7)	0 (0.0)	0 (0.0)
ALC on the day of IGRA, median (range), /μl	1480 (810–3190)	1640 (50–3980)	1990 (1110–2860)	1890 (800–3250)

Abbreviation: ATB, active tuberculosis; P-LTBI, patients with immunocompromised status and latent TB infection; H-LTBI, health care workers with latent TB infection; HC, health care workers confirmed with previous negative IGRA and low risks; PCR, polymerase chain reaction; TB, *M. tuberculosis*; ALC, Absolute lymphocyte counts.

**Table 2:** Distribution of QFT-Plus and TB-Feron IGRA results and agreement between the groups.

IGRA Patterns		Overall (%) (*n* = 335)	ATB (%) (*n* = 40)	P-LTBI (%) (*n* = 75)	H-LTBI (%) (*n* = 70)	HC (%) (*n* = 150)
Overall positivity of QFT-Plus		168 (50.1)	36 (90.0)	63 (84.0)	69 (98.6)	0 (0.0)
Overrall Positivity of TB-Feron		178 (53.1)	35 (87.5)	68 (90.7)	68 (97.1)	7 (4.7)
Q-Positive	T-Positive	164	34	63	67	0
TB1+ and TB2+		158 (96.4)	31 (91.2)	63 (100)	64 (95.5)	0 (0.0)
TB1 alone		2 (1.2)	0 (0.0)	0 (0.0)	2 (3.0)	0 (0.0)
TB2 alone		4 (2.4)	3 (8.8)	0 (0.0)	1 (1.5)	0 (0.0)
Q-Positive	T-Negative	4	2	0	2	0
TB1+ and TB2+		3 (66.7)	2 (100)	0 (0.0)	1 (50.0)	0 (0.0)
TB1 alone		0 (0.0)	0 (0.0)	0 (0.0)	0 (0.0)	0 (0.0)
TB2 alone		1 (33.3)	0 (0.0)	0 (0.0)	21(50.0)	0 (0.0)
Q-Negative	T-Positive	14	1	5	1	7
Q-Negative	T-Negative	151	3	5	0	143
Q-Negative	T-Indeterminate	2	0	2	0	0
Agreement of each group (%)		94.0	92.5	90.7	95.7	95.3

Abbreviation: IGRA, interferon-gamma-releasing assay; QFT-Plus and Q, QuantiFERON-TB Gold Plus; T, TB-Feron; TB1 or 2, TB antigen tube 1 or 2; ATB, active tuberculosis; P-LTBI, patients with immunocompromised status and latent TB infection; H-LTBI, health care workers with latent TB infection; HC, health care workers confirmed with previous negative IGRA and low risks.

**Table 3:** Results of IGRAs in 25 cases with discordant results between QFT-GIT/QFT-Plus and TB-Feron tests.

		Previous IGRA			Initial IGRA after Enrollment					Repeat IGRA during Follow Up				
No.	Group	QFT-GIT/QFT-Plus			QFT-Plus			TB-Feron		QFT-Plus			TB-Feron	
		TB1	TB2	Result	TB1	TB2	Result	TBF	Result	TB1	TB2	Result	TBF	Result
1	ATB				0.36	6.21	Positive	0.10	Negative					
2	ATB				0.35	0.97	Positive	0.08	Negative					
3	ATB				0.15	0.30	Negative	0.49	Positive					
4	P-LTBI	1.17	−	Positive	0.2	0.26	Negative	0.48	Positive					
5	P-LTBI	0.64	−	Positive	0.08	0.11	Negative	1.30	Positive					
6	P-LTBI	0.37	−	Positive	0.19	0.20	Negative	0.56	Positive	0.11	0.23	Negative	1.05	Positive
7	P-LTBI	0.49	0.66	Positive	0.16	0.06	Negative	0.38	Positive					
8	P-LTBI	0.26	0.40	Positive	0.06	0.08	Negative	0.43	Positive					
9	P-LTBI	0.37	−	Positive	0.02	0.03	Negative	0.05	Indeterminate ^†^					
10	P-LTBI	1.51	−	Positive	0.03	0.0	Negative	0.11	Indeterminate ^†^					
11	P-LTBI	1.79	−	Positive	0.01	0.0	Negative	0.07	Negative					
12	P-LTBI	0.53	0.62	Positive	0.01	0.01	Negative	0.04	Negative					
13	P-LTBI	0.35	0.33	Positive	0.01	0.04	Negative	0.20	Negative					
14	P-LTBI	0.49	0.60	Positive	0.08	0.07	Negative	0.07	Negative					
15	P-LTBI	1.28	1.01	Positive	0.0	0.0	Negative	0.0	Negative					
16	H-LTBI	>10	−	Positive	0.35	0.43	Positive	0.28	Negative					
17	H-LTBI	0.29	0.65	Positive	0.19	0.69	Positive	0.0	Negative	0.09	0.93	Positive	0.06	Negative
18	H-LTBI	0.42	−	Positive	0.14	0.18	Negative	0.43	Positive	0.06	0.13	Negative	0.48	Positive
19	HC				0.15	0.06	Negative	0.57	Positive	0.03	0.06	Negative	0.47	Positive
20	HC				0.05	0.12	Negative	0.69	Positive					
21	HC				0.002	0.13	Negative	0.82	Positive					
22	HC				0.02	0.0	Negative	>10	Positive					
23	HC	0.00	−	Negative	0.02	0.0	Negative	0.48	Positive	0.01	0.01	Negative	0.05	Negative
24	HC	0.15	−	Negative	0.0	0.34	Negative	1.11	Positive	0.20	0.0	Negative	0.01	Negative
25	HC	0.34	0.32	Negative	0.2	0.17	Negative	1.72	Positive	0.0	0.0	Negative	0.01	Negative

Previously performed QFT-GIT or QFT-Plus assays are presented as previous IGRA, and the QFT-Plus and TBF performed after enrollment were considered as initial and repeated QFT-Plus or TBF. ^†^ Indeterminate result due to mitogen minus nil <0.5. Absence of an IFN- value in the TB2 column for “Previous IGRA” presents the results from the QFT-GIT test. Abbreviations: IGRA, interferon-gamma-release assay; TB, tuberculosis; TB1, QFT-Plus TB1 tube; TB2, QFT-Plus TB2 tube; TBF, TB-Feron antigen tube; IFN-, interferon-gamma.

**Table 4:** Comparison of TB antigen-specific IFN-γ secreting T cell levels in response to TB antigens in the QFT-Plus and TB-Feron assays measured by flow cytometry intracellular cytokine analysis.

	No. of Cases	% of TB Antigen-Specific IFN- Secreting T Cells			*p*-Value ^†^ (TB1 vs. TB2)	*p*-Value ^†^ (TB1 vs. TBF)	*p*-Value ^†^ (TB2 vs. TBF)
		QFT-Plus TB1	QFT-Plus TB2	TB-Feron			
ATB CD4+	38	0.18 (0.11–0.29)	0.10 (0.04–0.21)	0.07 (0.02–0.23)	0.0308	0.2004	0.8777
ATB CD8+		0.10 (0.00–0.21)	0.04 (0.00–0.15)	0.22 (0.03–0.58)	0.3414	0.1597	0.0249
P-LTBI CD4+	10	0.12 (0.03–0.22)	0.11 (0.03–0.36)	0.22 (0.07–0.74)	0.2031	0.0645	0.0273
P-LTBI CD8+		0.00 (0.00–0.02)	0.00 (0.00–0.05)	0.34 (0.00–1.12)	0.6250	0.0469	0.0156
H-LTBI CD4+	8	0.06 (0.00–0.12)	0.06 (0.01–0.12)	0.02 (0.00–0.13)	0.8125	1.0000	0.6875
H-LTBI CD8+		0.00 (0.00–0.03)	0.00 (0.00–0.08)	0.21 (0.00–0.41)	0.6250	0.0625	0.0625
HC CD4+	12	0.02 (0.00–0.06)	0.02 (0.00–0.04)	0.00 (0.00–0.04)	0.7058	0.6166	0.5195
HC CD8+		0.00 (0.00–0.01)	0.00 (0.00–0.08)	0.19 (0.06–0.56)	0.1594	0.0304	0.0463

Percentage of IFN-γ released by CD4+ or CD8+ T lymphocytes, median (95% CI). ^†^ Categories in each group were compared using the Wilcoxon signed-rank test. Abbreviation: TB, tuberculosis; TB1 or 2, QFT-Plus TB antigen tube 1 or 2; TBF, Standard E TB-Feron; IFN-γ, interferon-gamma.
